# Hypomagnesemia in lymphoma patients receiving CAR T therapy correlates with immune dysfunction and decreased survival

**DOI:** 10.1186/s40164-025-00623-w

**Published:** 2025-04-30

**Authors:** Jennifer J. Gile, Patrizia Mondello, Zixing Wang, Ying Li, Radhika Bansal, Sangeetha Gandhi, Henan Zhang, Elham Babadi, Kodi Martinez, Gabrielle McCoy, Zuoyi Shao, Kevin Regan, Matthew A. Hathcock, Panwen Wang, Junwen Wang, Abdullah S. Al Saleh, Gordon Ruan, Stephen M. Ansell, N. Nora Bennani, Patrick B. Johnston, Jonas Paludo, Jose C. Villasboas-Bisneto, Arushi Khurana, Urshila Durani, Yucai Wang, Paul J. Hampel, Allison Rosenthal, Javier Munoz, Eider Moreno, Januario E. Castro, Hemant S. Murthy, Mohamed Kharfan-Dabaja, Saad S. Kenderian, Jenny J. Kim, Rhine Shen, Mike Mattie, Yi Lin, Thomas E. Witzig

**Affiliations:** 1 Division of Hematology, Mayo Clinic, 200 SW First Street, Rochester, MN 55905 USA; 2 Department of Hematology and Oncology, Mayo Clinic, Scottsdale, AZ USA; 3 Division of Hematology and Oncology, Mayo Clinic, Jacksonville, FL USA; 4 KITE, a Gilead Company, Santa Monica, CA 90404 USA; 5 Division of Experimental Pathology, Mayo Clinic, Rochester, MN 55905 USA

**Keywords:** Lymphoma, Magnesium, Cytokines, Survival analysis, CAR-T

## Abstract

**Background:**

Hypomagnesemia has been correlated with inferior outcomes in patients with large B cell lymphoma (LBCL) undergoing stem cell transplants. As T-cell and myeloid cell dysfunction have been associated with low magnesium conditions, we investigated whether serum magnesium (Mg) levels could predict clinical outcomes in LBCL patients who received chimeric antigen receptor T-cell therapy.

**Methods:**

Patients with LBCL who received axi-cel under the ZUMA-1 trial or as FDA approved therapy at Mayo Clinic were examined. Serum samples were obtained at specified time points and cytokine analysis was performed. Single cell RNA sequencing was performed on peripheral blood mononuclear cells. The Student T-test, Kruskal Wallis, or Fisher's Exact Tests were used to compare differences in demographics across Mg levels. Survival curves were plotted using the Kaplan–Meier methodology and compared using the Wilcoxon test.

**Results:**

We found that hypomagnesemia before lymphodepletion chemotherapy predicted inferior progression-free and overall survival in the pivotal study ZUMA-1 (NCT02348216). These results were validated in an independent cohort of LBCL patients receiving axicabtagene ciloleucel (axi-cel) at Mayo Clinic. Hypomagnesemia correlated with increased inflammatory serum markers and cytokine levels including ferritin, IL-6, IL1Ra, IL-8, and MIP1a. scRNAseq analysis unveiled altered immune interactions between monocytes and T cells with a concordant immune suppressive transcriptome.

**Conclusions:**

Hypomagnesemia at the time of CAR-T infusion is associated with an unfavorable inflammatory profile and decreased response and survival in LBCL patients receiving axi-cel. These findings suggest a potentially actionable prognostic factor for patients with large cell lymphoma undergoing CAR-T.

**Supplementary Information:**

The online version contains supplementary material available at 10.1186/s40164-025-00623-w.

## Introduction

Magnesium (Mg) is essential for regulating cell proliferation and immunity [1, 2]. Low serum Mg levels are common in hospitalized patients and associate with all-cause mortality [3]. Although monitoring serum Mg is commonly performed in patients undergoing platinum-based chemotherapy, there are no established guidelines for the target Mg level in oncologic patients as there are for those with cardiac conditions [4]. The relevance of serum Mg to lymphoma outcomes is derived from several lines of evidence. The discovery of X-linked immunodeficiency with Mg defect, EBV, and neoplasia (known as XMEN disease) suggests a role for Mg deficiency in the development of hematologic malignancies [5]. Mg influx to the intracellular space is essential to coordinate T cell activation. In patients with XMEN, a transport channel mutation impairs Mg influx, negatively affecting cytolytic responses. Recent data demonstrate that a novel gene editing approach in XMEN can restore Mg transporter expression and, in turn, the function of CD8^+^ T cells and natural killer cells [6]. Subsequent studies found that EBV viral loads were inversely related to plasma Mg levels suggesting that reduced Mg levels may impair T cell function, leading to uncontrolled viremia [7]. There are also several studies supporting the prognostic role of hypomagnesemia in patients with blood cancers. High posttransplant Mg levels have been associated with a lower incidence of relapse in patients with acute myeloid leukemia undergoing allogeneic stem cell transplant (SCT) [8]. We have reported that serum Mg levels below normal and even in the low normal range (1.7–1.9 mg/dL) at the time of autologous SCT for patients with relapsed diffuse large B cell lymphoma (DLBCL) is associated with an inferior progression-free survival (PFS) and overall survival (OS) post-transplant [9]. We also identified that hypomagnesemia at baseline is linked with inferior OS in patients with Burkitt Lymphoma [10].

Chimeric antigen receptor T-cell therapy (CART) is a relatively new cellular therapy where autologous T cells are engineered to express a chimeric antigen receptor targeting a cell surface antigen of interest (e.g., CD19) [11]. Based on the role of Mg in T-cell functions and our findings in SCT, we investigated whether serum Mg of patients with large B cell lymphoma (LBCL) at the time of lymphodepletion for CAR-T cells could influence their clinical response and outcome. We also explored the impact of hypomagnesemia on the composition and function of peripheral immune cells, which may partially explain prognosis. Since Axicabtagene ciloleucel (axi-cel, Yescarta) was the first FDA-approved and most used CAR-T therapy, we focused on LBCL patients receiving axi-cel in the registrational study ZUMA-1 (NCT02348216) and an independent patient cohort in our standard of care (SOC) practice at Mayo Clinic.

## Methods

### Clinical data and sample collection

Baseline demographics, cytokine release syndrome, neurotoxicity, clinical response, EFS, OS, clinical labs, and correlative blood samples for chemokines and cytokines were collected from subjects enrolled between 2015 and 2016 in cohorts 1 and 2 of the ZUMA-1 study [12, 13]. These patients are referred to as ZUMA-1 patients. Clinical data from the medical records of patients with LBCL who received SOC axi-cel between January 2018 and May 2020 at Mayo Clinic Rochester, MN, were reviewed after the Mayo Clinic Investigational Review Board (IRB) approval. They are referred to SOC patients. Serum Mg levels (normal range, 1.7–2.3 mg/dL) were collected on day -5 before the start of LD chemotherapy. Baseline demographics, clinical response, disease progression date, and date of death were collected. Lugano lymphoma response criteria were used to assess clinical response [14]. Additional blood was assayed for single-cell RNA sequencing for a subset of SOC subjects who consented to a Mayo IRB-approved biobank protocol.

### Cytokine analysis from blood

Serum samples were obtained on Day -5 (pre-lymphodepletion), Day 0 (post-conditioning and pre-axi-cel infusion) and at peak CAR-T expansion (typically day 7 or 14). A panel of analytes representing major categories of immune function were measured in serum by Meso Scale Discovery®. Spearman correlation analysis was performed to examine the relationship between cytokine levels at the indicated timepoints with baseline Mg level.

### PBMNC isolation and scRNAseq

Blood samples were available from 13 sequentially relapsed/refractory LBCL patients receiving CART cell therapy and enrolled to the biobank protocol which had either blood Mg level above 2.0 mg/dL and had durable complete remission for at least six months or more (Mg^high^) or Mg level below 2.0 mg/dL and had progressive disease within six months (Mg^low^) were used for scRNAseq. Peripheral blood mononuclear cells (PBMNC) were isolated from heparinized blood samples using Lymphoprep (Stemcell Technologies) density gradient centrifugation according to the manufacturer's instructions and cryopreserved in CryoStor CS10 (BioLife Solutions, Inc.). On the day of the single cell collection for RNA-sequencing, the cells were thawed for 1 min and washed with buffer (PBS with 2% BSA and 2 mM EDTA). PBMNC were counted and processed immediately for single-cell capture and 5' RNA-sequencing (10X Genomic). Gene expression libraries were prepared for each sample according to the manufacturer's protocol (10X Genomics). All libraries were sequenced using 10,000 cells to achieve a minimum of 5,000 cell reads per cell for gene expression.

### scRNAseq analysis

Single-cell RNA sequencing (scRNA-Seq) data are aligned and quantified using 10X Genomics Cell Ranger Software Suite (v4.0) against the human reference genome (hg38). Supervised single-cell analysis was performed by mapping scRNA-seq datasets to the annotated human PBMC reference using the Seurat package (v4.0) [15] with SCTransform normalization and default parameters for the FindTransferAnchors and MapQuery functions. Cells with fewer than 200 detected genes, > 40,000 or < 500 total UMI counts, or > 25% mitochondrial genes were excluded from subsequent analyses. Differential gene expression analysis was performed between the CR High-Mg group and PD Low-Mg group at cluster level at different timepoints using the FindMarkers function by Seurat. The differentially expressed gene lists were used for GSEA (v4.2.3) pre-ranked analysis using − log10(p-value)*log2FC as the ranking score against the MSigDB Hallmark Gne Sets. CellChat (v1.5.0, Vu et al. [16]) was used to perform cell–cell-interaction analysis separately for each condition at each timepoint by identifying over-expressed ligands or receptors among each cell subpopulation, followed by identifying over-expressed ligand-receptor interactions [15, 16]. Biologically significant cell–cell communication was inferred by a probability value assigned to each interaction through integrating gene expression with CellChatDB, a manually curated database of known interactions between signaling ligands, receptors, and their cofactors. Cell–cell interactions between Mg^high^ and Mg^low^ groups were compared for each timepoint.

### Statistical analysis

The Student T-test, Kruskal Wallis, or Fisher's Exact Tests were used to compare differences in demographics across Mg levels. To assess clinical outcomes, we used the following parameters: Progression-free survival (PFS)—defined from the time of CAR-T infusion to relapse, disease progression, or death of any cause; Event-free survival (EFS)—defined from the time of CAR-T infusion to relapse, disease progression, start of another lymphoma directed therapy or death of any cause, whichever occurred first; OS was defined from the time of CAR-T infusion to death of any cause. Survival curves were plotted using the Kaplan–Meier methodology and compared using the Wilcoxon test. Additional analysis between MG and CRP, Ferritin, and Cytokines were analyzed using Pearson Correlation Coefficient. A p < 0.05 was considered statistically significant (two-tailed testing). All analysis was performed with R (Version 3.6.3, R Foundation for Statistical Computing, Vienna, Austria).

## Results

### Patient characteristics

We included 108 patients from cohort 1 (enrolling DLBCL) and cohort 2 (enrolling primary mediastinal B cell lymphoma and transformed follicular lymphoma) of the ZUMA-1 clinical trial and 57 LBCL patients from an independent cohort who received axi-cel as SOC at Mayo Clinic. Demographics are shown in Tables 1 and 2, respectively.

**Table 1:** Patient characteristics stratified by magnesium level in ZUMA-1 cohort

	Low Mg (< 1.7 mg/dL) N = 23	Normal Mg (1.7 – < 2.0 mg/dL) N = 48	High Mg (≥ 2.0) N = 37	Total N = 108	P value
Age					
Median (Q1, Q3)	56 (47, 67)	57 (51, 64)	59 (55, 64)	58 (51, 65)	0.59
Range	25–75	23–76	28–76	23–76	
Male sex	15 (65%)	30 (63%)	28 (76%)	73 (68%)	0.42
Caucasian race	19 (83%)	40 (83%)	33 (89%)	92 (85.2)	0.70
Diagnosis					
DLBCL	19 (83%)	33 (69%)	32 (87%)	84 (78%)	0.18
HG					
PMBCL	0 (0%)	6 (13%)	2 (5%)	8 (7%)	
TFL	4 (17%)	9 (19%)	3 (8%)	16 (15%)	
ECOG PS ≥ 1	16 (70%)	32 (67%)	14 (38%)	62 (57%)	0.01
Elevated LDH (> 190 U/L), no (%)	22 (96%)	39 (81%)	32 (87%)	93 (86%)	0.26
IPI ≥ 3, number (%)	12 (52.2%)	20 (41.7%)	17 (45.9%)	49 (45.4%)	0.71
Stage III/IV no (%)	19 (82.6%)	37 (77.1%)	34 (91.9%)	90 (83.3%)	0.19
Prior Lines of Therapy					0.426
Median (Q1, Q3)	4 (2, 4)	3 (2, 4)	3 (2, 4)	3 (2, 4)	
Range	1—10	1–8	2–6	1–10	
Prior ASCT	6 (26%)	13 (27%)	10 (27%)	29 (26.9%)	> 0.99

Mg: magnesium; DLBCL: diffuse large B cell lymphoma; HG: high-grade lymphoma; PMBCL: primary mediastinal large B cell lymphoma; TFL: transformed follicular lymphoma; ASCT: autologous stem cell transplant; ECOG: PS eastern cooperative oncology group performance status; LDH: lactate dehydrogenase; IPI international prognostic index; LD: chemo lymphodepleting chemotherapy

**Table 2:** Patient characteristics stratified by magnesium level in SOC Cohort

	Low Mg (< 1.7 mg/dL) N = 12	Normal Mg (1.7 – < 2.0 mg/dL) N = 30	High Mg (≥ 2.0) N = 15	Total N = 57	P value
Age					
Median (Q1, Q3)	61 (55, 65)	57 (41, 62)	60 (54, 64)	59 (44–64)	0.20
Range	38–75	27–77	27–77	27–77	
Male sex	6 (50%)	18 (60%)	10 (67%)	34 (60%)	0.67
Caucasian race	8 (67%)	26 (87%)	13 (87%)	47 (82%)	0.33
Diagnosis					
DLBCL	5 (42%)	22 (73%)	9 (60%)	36 (63%)	0.14
HG	4 (33%)	2 (7%)	1 (7%)	7 (12%)	
PMBCL	0 (0%)	0 (0%)	1 (7%)	1 (2%)	
TFL	3 (25%)	6 (20%)	4 (27%)	13 (23%)	
ECOG PS ≥ 1	6 (50%)	11 (37%)	6 (40%)	23 (40%)	0.72
Elevated LDH (> 220 U/L), no (%)	9 (75%)	19 (63%)	10 (67%)	38 (67%)	0.87
IPI ≥ 3, number (%)	9 (75%)	13 (43%)	9 (60%)	31 (54%)	0.18
Stage III/IV Yes (%)	12 (100%)	27 (90%)	15 (100%)	54 (95%)	0.41
Extranodal disease	11 (92%)	16 (53%)	10 (67%)	37 (65%)	0.07
Prior lines of therapy					
Median (Q1, Q3)	3.5 (3, 4)	3 (3, 4)	3.0 (2–4)	3.0 (3, 4)	0.25
Range	3–5	1–6	2–5	1–6	
Prior ASCT	6 (50%)	14 (47%)	5 (33%)	25 (44%)	0.68

Mg: magnesium; DLBCL: diffuse large B cell lymphoma; HG high-grade lymphoma; PMBCL: primary mediastinal large B cell lymphoma; TFL transformed follicular lymphoma; ASCT: autologous stem cell transplant; ECOG PS: eastern cooperative oncology group performance status; LDH lactate dehydrogenase; IPI: international prognostic index; LD: chemo lymphodepleting chemotherapy

The two groups were similar regarding age, diagnosis, and sex. There were minor differences in the percent of prior autologous stem cell transplant (ASCT), performance status (PS), and abnormal LDH levels. The median serum Mg levels before lymphodepletion chemotherapy (LD) were similar between the two cohorts (1.9 mg/dL; range, 1.2–2.4 mg/dL in the ZUMA-1 cohorts and 1.9 mg/dL; range, 1.3–2.3 in the Mayo Clinic cohort). Mg levels from the start of LD chemotherapy through day 28 post-CAR-T infusion remained consistent (Supplemental Fig. 1).

For analysis purpose (Tables 1 and 2), we divided patients in 3 groups based on Mg levels: low Mg (< 1.7 mg/dL [Mg^low^]), normal Mg ($\ge$ 1.7 < 2.0 mg/dL [Mg^NL^]) and high (optimal) Mg ($\ge$ 2 mg/dL [Mg^high^]). These cutoffs were chosen based on prognostic values identified on our prior work^9,10^. In the ZUMA-1 cohorts, 21.3% (23/108) subjects were Mg^low^, 44.4% (48/108) subjects Mg^NL^, and 34.3% (34/108) subjects were in the Mg^high^ range. The Mayo Clinic cohort distribution was similar, with 21% (12/57) Mg^low^, 53% (30/57) Mg^NL^, and 26% (15/57) Mg^high^. There was no difference in patient characteristics among the groups with different Mg levels except for a worse PS in those with Mg^low^ compared Mg^high^ in ZUMA-1 cohort (70% vs 38%, p = 0.01). However, this difference was not evident in the Mayo Clinic cohort (50% vs 40%, p = 0.72).

### Magnesium level correlates with clinical response and survival

**Fig. 1:**
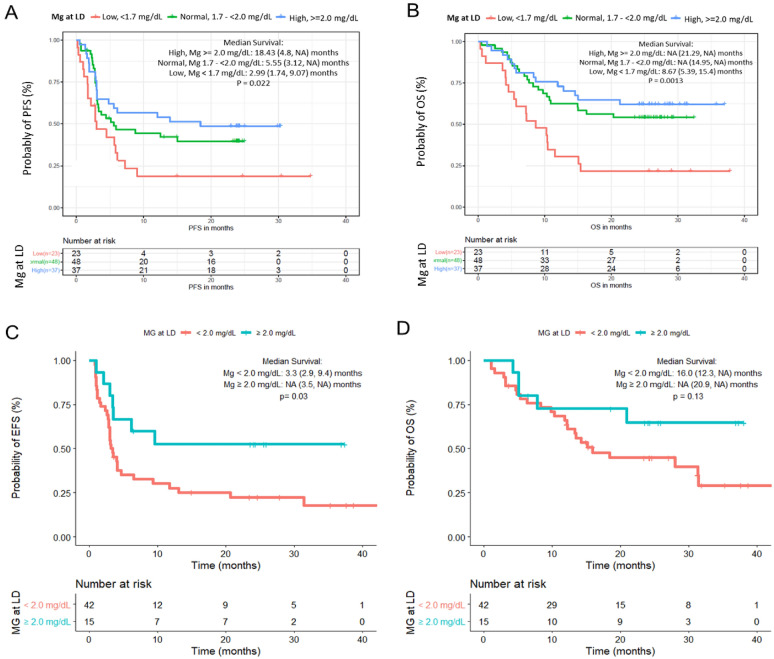
Survival grouped by magnesium level. **A** Progression-free survival of patients with lymphoma in the ZUMA-1 cohort undergoing CAR-T by day -5 before the start of lymphodepleting chemotherapy serum magnesium level. p = 0.022. **B** Overall survival of patients with lymphoma in the ZUMA-1 cohort undergoing CAR-T by day -5 before the start of lymphodepleting chemotherapy serum magnesium level. p = 0.0013. (Low ≤ 1.7 mg/dL; Normal = 1.7 to < 2.0 mg/dL; High ≥2.0 mg/dL) **C** Progression-free survival of patients with lymphoma in the SOC cohort undergoing CAR-T by day -5 before the start of lymphodepleting chemotherapy serum magnesium level. p = 0.03. **D** Overall survival of patients with lymphoma in the SOC cohort undergoing CAR-T by day -5 before the start of lymphodepleting chemotherapy serum magnesium level. p = 0.13

In the ZUMA-1 cohort, there was a lower overall response rate (ORR) in patients with hypomagnesemia (65%, 15/23) compared to those with normal (83%, 40/48) and high Mg levels (92%, 34/37; p = 0.03). This translated to an inferior PFS (p = 0.022; Fig. 1A) and OS (p = 0.0013; Fig. 1B). In the Mayo Clinic cohort, due to the smaller number of patients by tertile, we examined clinical response and survival using Mg < or ≥ 2.0 mg/dL grouping. The PFS and OS by tertile grouping of Mg levels are shown in Supplemental Fig. 2A and 2B.

This is a commonly used clinical threshold for magnesium replacement in patients with cardiac issues.[17] Fitting a spline of the partial residuals for Mg at LD shows clinically significant benefit for Mg at 2.0 mg/dL and higher as a reasonable cut point. (Supplemental Fig. 2 C, D).

**Fig. 2:**
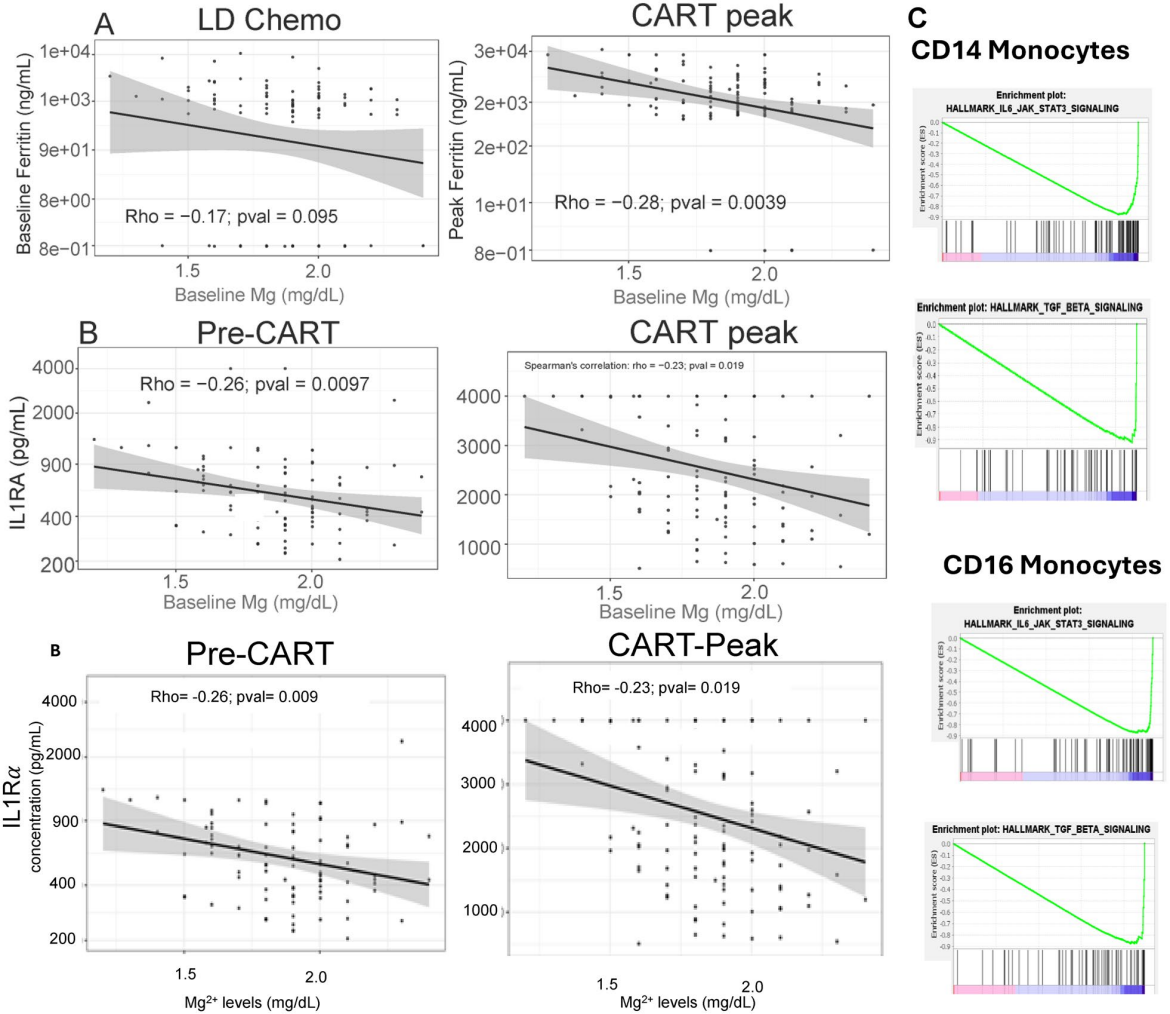
Correlation of magnesium level with inflammatory markers and monocyte transcriptome. **A** Magnesium level at the time of lymphodepletion chemotherapy (Mg at LD Chemo) and ferritin at LD chemo, CART infusion, and CART peak levels (column labels) are shown for ZUMA-1 study (top row) and Mayo SOC cohort (bottom row). **B** Mg level prior to CART (x-axis) and cytokine IL1RAare shown for time points as labeled for each column for ZUMA-1 study. (**A** and **B** Lines represent linear regression. The shaded region represents a 95% confidence interval. Pearson correlation and p-value are noted in the graph.) **C** GSEA analysis for enriched pathways is shown for CD14 + monocytes and CD16 + monocytes, comparing patients with Mg^High^ (Mg ≥ 2.0 mg/dL) and with Mg^low^ (Mg < 2.0 mg/dL)

The complete response (CR) rate trended higher in patients with Mg ≥ 2.0 mg/dL (80% v 52%; p = 0.07). Event free survival (EFS) was examined in the Mayo Clinic cohort due to some patients with stable disease or partial response receiving the next line of lymphoma-directed treatment before meeting Lugano's criteria for progressive disease. The patients with low Mg levels confirmed an inferior EFS (HR 0.41, 95% CI 0.18–0.93, p = 0.034; Fig. 1C), while the OS did not achieve significance (HR 0.49, 95% CI 0.17–1.28, p = 0.13; Fig. 1D) probably due to the low power and shorter follow-up. The median follow-up for ZUMA-1 was 15.4 months, and for the Mayo Clinic SOC cohort was 14 months.

### Magnesium levels inversely correlate with inflammatory markers and immune effector cell-associated toxicities

Levels of C-reactive protein (CRP) (data not shown) and ferritin, which are common inflammatory markers, were inversely correlated with Mg levels in both the ZUMA-1 and Mayo SOC cohorts (Fig. 2A). In addition, in the ZUMA-1 cohort, Mg levels were inversely correlated with the levels of IL1Ra, IL6 and MIP1a before LD and at CART cell peak (Fig. 2B), suggesting an impairment of immune function.

Given the known contribution of myeloid cells to these cytokines and chemokines, we examined the monocyte transcriptomes at CAR-T peak expansion. We found that CD14 and CD16 monocytes from patients with Mg^low^ levels were enriched for IL-6/STAT3 and TGFb gene pathways compared to those from patients with Mg^high^ levels (Fig. 2C).

**Fig. 3:**
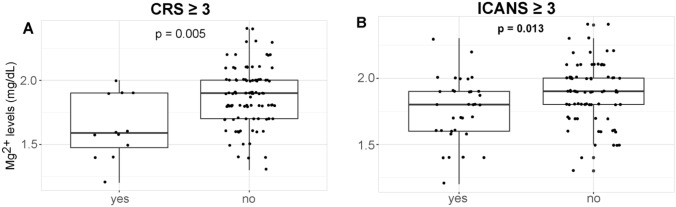
Cytokine release syndrome and neurotoxicities by magnesium level. Magnesium levels for patients in the ZUMA-1 study at the time of lymphodepletion chemotherapy (Mg at LD) are shown for patients experiencing grade 3 or higher cytokine release syndrome (CRS) (**A**) and neurotoxicity (**B**). ASTCT guideline was used for CRS and neurologic events grading in the SOC cohorts

Patients in ZUMA-1 with hypomagnesemia at LD-chemo were more likely to develop grade 3 or higher cytokine release syndrome (CRS) and neurologic events (Fig. 3A, B) as graded by Lee's criteria [18]. In the Mayo SOC cohort, we did not observe a significant difference in the severity of CRS and Immune cell associated neurotoxicity syndrome (ICANS) among the Mg groups likely due to improved CRS and ICANS management practices that have evolved since ZUMA-1 [19].

### Tight link between magnesium levels and immune cell interactions

**Fig. 4:**
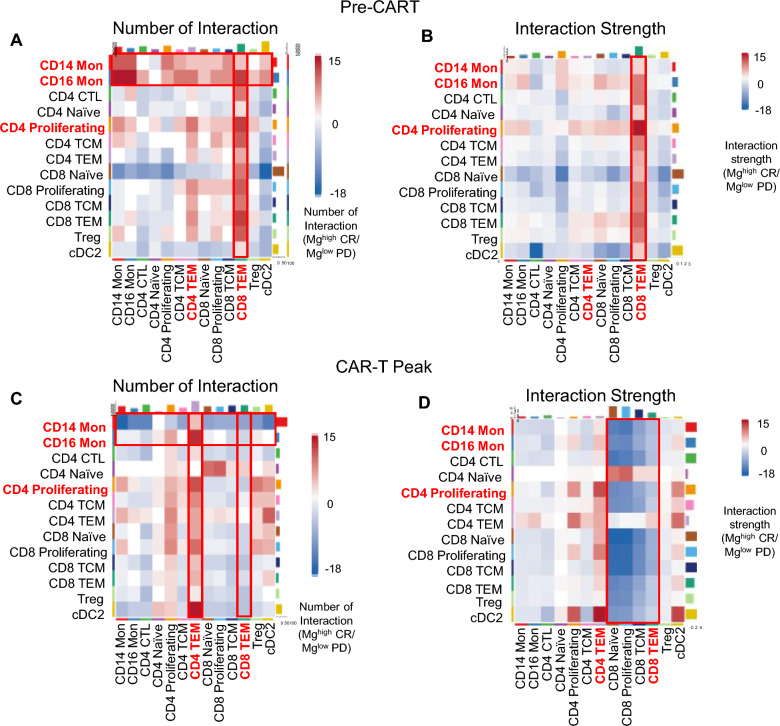
Immune cell interactions by magnesium levels. Heatmap showing the differential number of interactions (**A**) and strength of interactions (**B**) among the indicated immune cells at CAR-T infusion and at CAR-T peak (**C**, **D**)

To explore whether the levels of Mg impact systemic immune cells, we performed single-cell RNA-sequencing (scRNA-seq) of available peripheral blood cells collected from 13 LBCL patients from the Mayo SOC cohort before LD chemotherapy (Pre-CART) and at peak CAR-T expansion (CART-Peak). Using CellChat analysis, we found that at Pre-CART, patients with Mg^high^ levels had a higher number and strength of interactions between CD16 and CD14 monocytes and between these cells with CD4 and CD8 T cells compared to those with Mg^low^ levels (Fig. 4A and B). Similarly, CD8 T effector memory (T_EM_) cells had an increased number and strength of interactions with all other immune cells in Mg^high^ patients (Fig. 4A), with the maximal interaction strength evident between CD8 T_EM_ cells and CD4 proliferating cells, followed by CD16 monocytes (Fig. 4B).

**Fig. 5:**
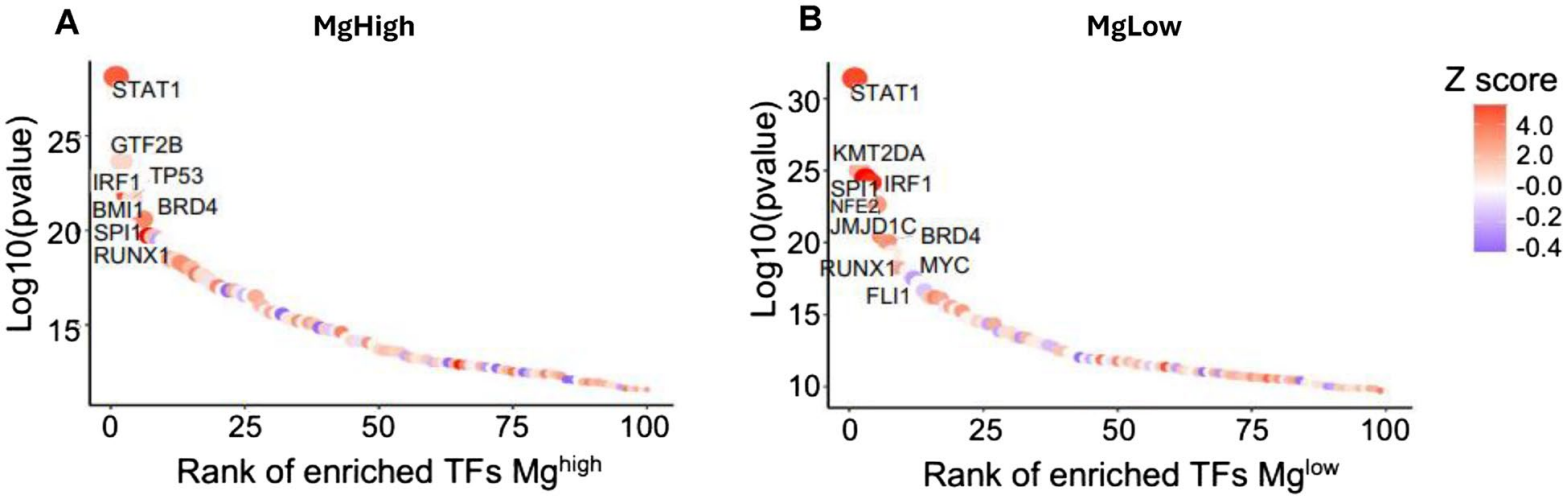
Transcription factor analysis by magnesium levels. Transcription factor analysis identifying the factors significantly expressed at CART-Peak in patients with Mg^high^ (**A**) and those with Mg^low^ (**B**)

Remarkably, at CART-Peak, the same interactions decreased dramatically in patients with Mg^high^ (Fig. 4C and D). There was a profound decrease in the overall interaction strength of all CD8 T cell subsets, including CD8 naïve, proliferating, central memory (T_CM_), and T_EM_ cells in the Mg^high^ group (Fig. 4D). In contrast, we observed a substantial upregulation of the CD4 T_EM_ cells, suggesting the presence of a sustained antigen-restricted immune response. A transcription factors enrichment analysis identified significant enrichment for *SPI1* (also known as *PU.1*) in Mg^low^ patients compared to those Mg^high^. The transcription factor *SPI1* plays an essential role in monocyte differentiation [20, 21] and reprograms macrophages towards suppressive M2 subtype [22]. There was also an enrichment of *KMT2A* (also known as *MLL1*), a histone methyltransferase that regulates mono- and dimethylation of histone 3 at lysine 4 (H3K4me1/2) primarily at gene enhancers. This epigenetic change may alter the chromatin accessibility to allow transcription factor binding (e.g., *SPI1*) and gene activation. Along the same line, *JMJD1C* (also known as *KDM3C*) significantly increased in Mg^low^ patients who progressed. *KDM3C* belongs to an enzymatic family that specifically demethylates H3K4me1/2 resulting in suppression [23] of the NF-kB-mediated immune response [24]. Moreover, Mg^low^ non-responders showed an upregulation of the proto-oncogene *MYC*, which favors stemness and cell growth as well as orchestrates changes in the TME, including immune suppression[25] (Fig. 5). Finally, we performed a ligand-receptor analysis to identify potentially unique inhibitory cell–cell communication mechanisms in each immune subset at CART cell peak. Interestingly, we found a significant downregulation of multiple HLA subtypes linked to CD8 T cells (naïve, proliferating, T_CM,_ and T_EM_) interacting with CD14 and CD16 monocytes, CD4 proliferating cells, CD4 and CD8 T_EM_ cells in Mg^high^ responders. By contrast, the same interactions were significantly increased in the Mg^low^ progressors. The results of colocalized ligand-receptor pairs and cell-specific markers infer a signaling network facilitating contact-dependent communication and immune response. Altogether, these data underscore the relevance of Mg in modulating the intercellular interaction for successful CART cell function.

## Discussion

Magnesium plays a critical role in modulating immune surveillance against pathogens and cancer cells. Previous studies have shown a faster rate of tumor growth and a lower infiltration of T cells in vivo models of hypomagnesemia. Additionally, the relevance of Mg levels has shown remarkable implication in immunotherapeutic strategies in B cell lymphoma including checkpoint inhibitors and autologous SCT, however the impact of Mg on CART cell therapy in humans remains underexplored. To answer this question, we leveraged two independent patient cohorts including the ZUMA-1 trial and our Mayo Clinic SOC cohort. Our data demonstrate the prognostic impact of Mg in relapsed/refractory LBCL patients receiving axi-cel CAR-T therapy and uncovered its functional implication in immune response.

Interestingly, we found that low levels of Mg prior to the start of lymphodepletion (LD) chemotherapy were associated with increased expression of inflammatory markers such as CRP and ferritin. These inflammatory markers are also known to be prognostic for poor response and decreased survival [26]. In addition, hypomagnesemia is associated with decreased presence of T and NK cells and increased levels of myeloid cell-associated inflammatory cytokines and chemokines. Myeloid-derived cytokines are a driver of CAR-T-associated toxicities, CRS, and ICANS. Correspondingly, patients in the ZUMA-1 study with more severe CRS and ICANS have been found to display hypomagnesemia at the time of lymphodepletion. However, this correlation was not confirmed in the Mayo Clinic cohort, likely due to changes in CRS management and the lower incidence of severe CRS in SOC practice [27, 28].

There is minimal data on serum Mg levels status in lymphoma correlated with clinical outcomes in general and in the setting of CAR-T cell therapy specifically. We have previously demonstrated that in patients with newly diagnosed diffuse large B cell lymphoma, only 3% had a low serum Mg. Previous CAR-T trials have documented that 19% of patients in the phase 1 axi-cel trial [13] experienced hypomagnesemia, and all but 1 were grade 1. The study with lisocabtagene maraleucel [29] also reported 19% hypomagnesemia, with all being ≤ grade 2. Thus, low serum Mg levels appear to worsen as patients receive more treatments. Neither of these studies reported outcomes by Mg level status. Of note, grade 1 hypomagnesemia (levels between 1.2 annd 1.6 mg/dL) may be considered a "mild toxicity" but was associated with poor outcomes in our study. We previously reported in a much larger study of relapsed DLBCL patients undergoing a standard autologous SCT that serum Mg levels < 2.0 mg/dL predicted an inferior outcome [9]. Based on our studies of Mg in relapsed DLBCL patients undergoing autologous SCT, and the previous studies that hypomagnesemia leads to impaired T cell responses [30–32], we hypothesized that patients with hypomagnesemia undergoing CAR-T cell therapy would have inferior outcomes. Taken together, our data in the registration ZUMA-1 study and our SOC patients confirm that hypomagnesemia is a prognostic factor and predictor of inferior PFS/EFS compared to patients with high normal Mg levels at this timepoint.

It has long been known that effective T-cell function requires Mg [33, 34]. Specifically, the lymphocyte function-associated antigen 1 (LFA-1), a T cell surface protein required for the formation of the immune synapse, necessitates Mg for its conformation and ability to activate T cells [35]. This was further validated recently by Lötscher et al. [36] who demonstrated the importance of extracellular Mg to CD8 T-cell function via LFA-1 signaling. They showed that hypomagnesemia impaired response to checkpoint inhibitor therapy in lung cancer patients. They also retrospectively reviewed 96 patients treated with axi-cel in the SOC practice and found that those with hypomagnesemia around the time of CAR-T treatment (using a level < 1.7 mg/dL) had an inferior outcome. Our data further identifies altered monocyte transcriptomes, including enrichment of cytokine pathways such as IL-6 and TGFb, which are known to be involved in unfavorable inflammation[37] and immune suppression[38, 39]. In addition, altered monocyte interaction with T cells may further contribute to the correlation of hypomagnesemia with decreased PFS. Our findings come from a larger cohort of patients in both the registration clinical trial and SOC practice.

The strengths of our study include the standard of care cohort that is used as confirmation of the findings in the ZUMA-1 cohort. The SOC cohort was all performed at one institution with the same CAR-T team evaluating each patient and as a result, there was a unified approach to patient selection and treatment. We have also found similar outcomes in patients with large cell lymphoma undergoing autologous stem cell transplant, further strengthening these results. in addition, we have identified a modifiable nutritional biomarker that is supported by the science that Mg is essential for T-cell function. Magnesium replacement is a low-cost approach to further improve CAR-T outcomes. Finally, we found that patients with optimal Mg levels had a better ECOG performance status than those with low serum Mg level, this further supports the idea that patients with higher serum Mg levels have better outcomes.

Mg replacement could be a readily available intervention to facilitate a favorable environment for the activities of the infused CAR-T cells. However, replacing Mg in ill patients can be difficult as these patients typically do not eat well and may waste Mg through the GI tract (diarrhea) and kidney (due to prior platinum-based chemotherapy [40] and anti-fungals such as amphotericin [41]. In addition, certain bridging chemotherapeutic agents can lead to low serum magnesium levels.

Laboratory serum Mg deficiency at our institution is defined as a Mg level < 1.7 mg/dL. However, this study found a cut-off of < 2.0 mg/dL was associated with poor response rate and EFS. This is the same level we found to be prognostic in the DLBCL SCT study [9]. In addition, values less than 2.0 mg/dL are associated with an increased risk of congestive heart disease mortality [42] and sudden cardiac death [43] in patients with cardiac pathologies. In patients with a risk of cardiac arrhythmia and other events, it is common practice to keep the Mg level at ≥ 2.0 mg/dL. Although there are other prognostic factors for CAR-T outcomes, magnesium is one of the few that is actionable. However, we acknowledge that it is unknown whether replacing Mg to a value of ≥ 2.0 mg/dL at time of LD chemotherapy will lower the risk of CRS and improve overall outcomes to CART cell therapy.

## Conclusions

This study provides insight into the clinical relevance of hypomagnesemia in patients with lymphoma undergoing CAR-T therapy. Our analysis has similar demographics and percentages of patients with hypomagnesemia compared to other published clinical trials on CAR-T [13, 29]. However, we acknowledge the limitation of our study concerning its relatively small sample size and retrospective design, in addition we do not have granular data on the specifics of any bridging chemotherapy that was given prior to CAR-T which may be another contributor to hypomagnesemia. Regardless of the etiology for hypomagnesemia, we believe the next step is to study whether correction of serum Mg level ≥ 2.0 mg/dL will reduce inflammation and improve outcomes. We anticipate that prospective studies evaluating the immune cell dysfunctions associated with hypomagnesemia will shed light into intervention strategies that can improve the outcome of patients treated with cellular therapies.

## Supplementary Information

Supplementary Material 1: Supplemental Figure 1. Magnesium level over time. Magnesium levels from the start of LD chemotherapy through day 28 post-CAR-T infusion are shown for patients in the ZUMA-1 study (top panel) and the SOC cohort (bottom panel). The solid line represents the median magnesium level and the dotted lines represent the interquartile ranges.

Supplementary Material 2: Supplemental Figure 2. Survival by magnesium level for SOC cohort. A. Progression-free survival of patients with lymphoma in the SOC cohort undergoing CAR-T by day -5 before the start of lymphodepleting chemotherapy serum magnesium level. p=0.058. B. Overall survival of patients with lymphoma in the SOC cohort undergoing CAR-T by day -5 before the start of lymphodepleting chemotherapy serum magnesium level. p=0.032. The line color indicates magnesium level grouping. Spline plots for the relative hazard ratio are shown for EFS (C) and OS (D). The dotted lines correspond to a 95% confidence interval.

## Data Availability

No datasets were generated or analysed during the current study.

## Abbreviations

LBCL: Large B cell lymphoma
Mg: Magnesium
Axi-cel: Axicabtagene ciloleucel
XMEN: X-linked immunodeficiency with Mg defect, EBV and neoplasia
SCT: Stem cell transplant
PFS: Progression-free survival
OS: Overall survival
CAR-T: Chimeric antigen receptor T-cell
SOC: Standard of care
IRB: Investigational review board
EFS: Event free survival
ASCT: Autologous stem cell transplant
PS: Performance status
ORR: Overall response rate
LD: Lymphodepletion chemotherapy
CR: Complete response
CRP: C-reactive protein
CRS: Cytokine release syndrome
ICANS: Immune cell associated neurotoxicity syndrome
ASTCT: American society of transplant cell therapy
scRNA-seq: Single-cell RNA-sequencing
LFA-1: Lymphocyte function-associated antigen 1
