# Supplementary figures and images for: Hypomagnesemia in lymphoma patients receiving CAR T therapy correlates with immune dysfunction and decreased survival
Source: Exp Hematol Oncol. 2025 Apr 30;14:63. doi: 10.1186/s40164-025-00623-w (PMC12044716; doi:10.1186/s40164-025-00623-w)

## Slide 1
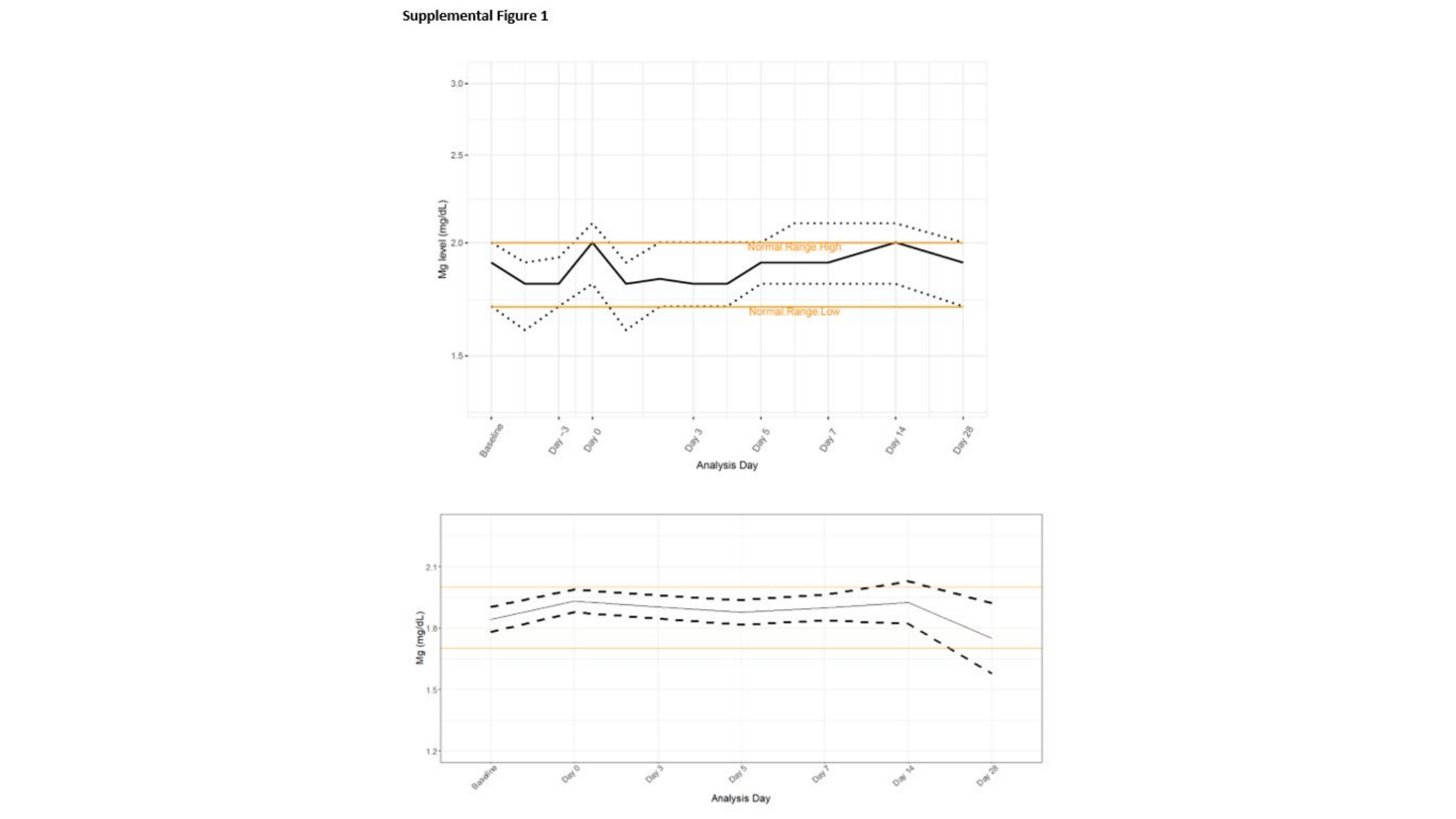

Supplement: Supplementary file 1 — Supplementary Material 1: Supplemental Figure 1. Magnesium level over time. Magnesium levels from the start of LD chemotherapy through day 28 post-CAR-T infusion are shown for patients in the ZUMA-1 study (top panel) and the SOC cohort (bottom panel). The solid line represents the median magnesium level and the dotted lines represent the interquartile ranges. [file 40164_2025_623_MOESM1_ESM.pptx]

## Slide 1
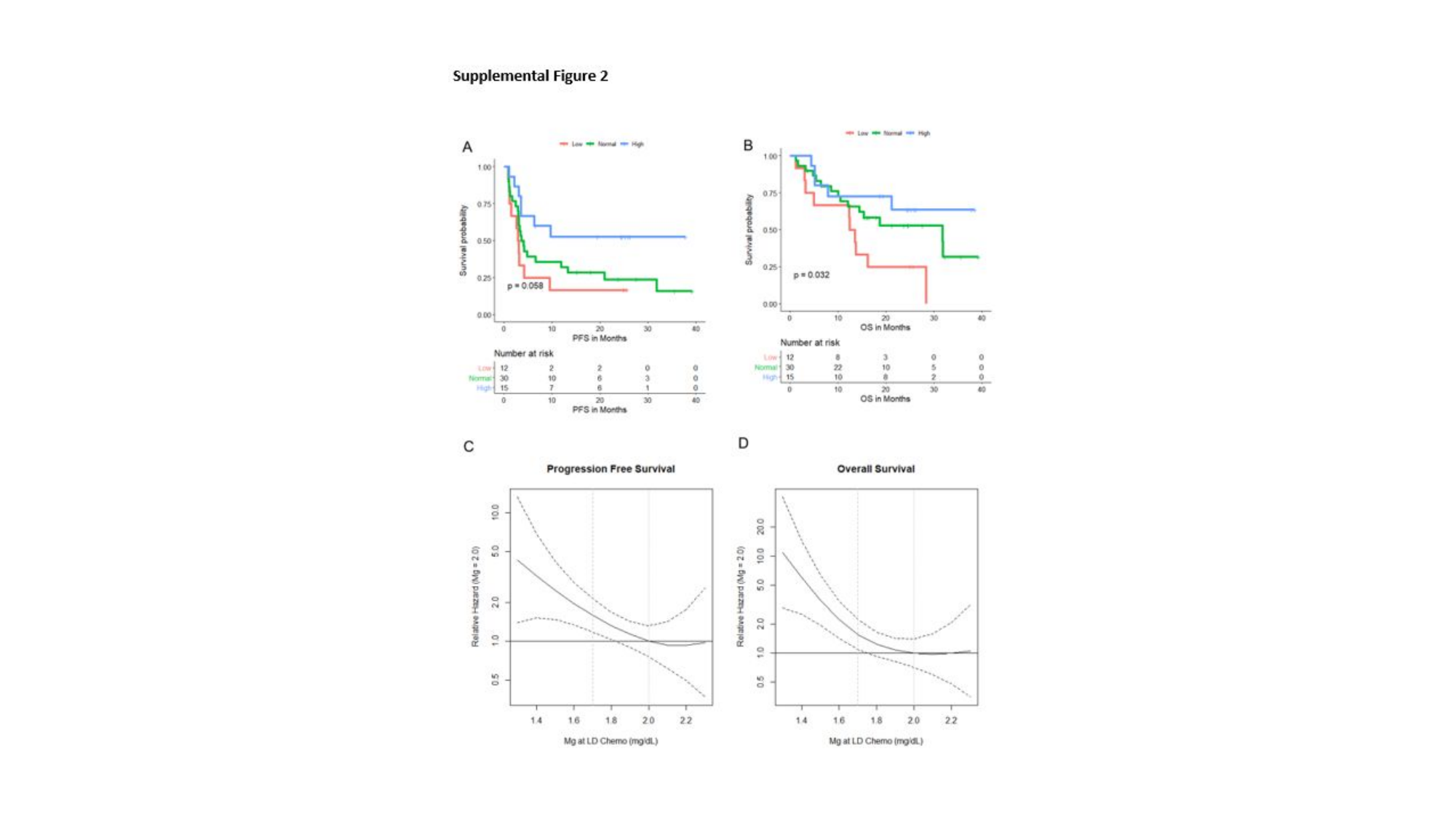

Supplement: Supplementary file 2 — Supplementary Material 2: Supplemental Figure 2. Survival by magnesium level for SOC cohort. A. Progression-free survival of patients with lymphoma in the SOC cohort undergoing CAR-T by day -5 before the start of lymphodepleting chemotherapy serum magnesium level. p=0.058. B. Overall survival of patients with lymphoma in the SOC cohort undergoing CAR-T by day -5 before the start of lymphodepleting chemotherapy serum magnesium level. p=0.032. The line color indicates magnesium level grouping. Spline plots for the relative hazard ratio are shown for EFS (C) and OS (D). The dotted lines correspond to a 95% confidence interval. [file 40164_2025_623_MOESM2_ESM.pptx]
